# Antiproliferative and Pro-Apoptotic Effect of Uvaol in Human Hepatocarcinoma HepG2 Cells by Affecting G_0_/G_1_ Cell Cycle Arrest, ROS Production and AKT/PI3K Signaling Pathway

**DOI:** 10.3390/molecules25184254

**Published:** 2020-09-16

**Authors:** Gloria C. Bonel-Pérez, Amalia Pérez-Jiménez, Isabel Gris-Cárdenas, Alberto M. Parra-Pérez, José Antonio Lupiáñez, Fernando J. Reyes-Zurita, Eva Siles, René Csuk, Juan Peragón, Eva E. Rufino-Palomares

**Affiliations:** 1 Department of Biochemistry and Molecular Biology I, Faculty of Sciences, University of Granada, Avenida Fuentenueva, 1, 18071 Granada, Spain; gloria1797@correo.ugr.es (G.C.B.-P.); isagc@ugr.es (I.G.-C.); ampperez@correo.ugr.es (A.M.P.-P.); jlcara@ugr.es (J.A.L.); ferjes@ugr.es (F.J.R.-Z.); 2 Department of Zoology, Faculty of Sciences, University of Granada, Avenida Fuentenueva, 1, 18071 Granada, Spain; calaya@ugr.es; 3 Department of Experimental Biology, University of Jaen, Campus Las Lagunillas s/n. 23071 Jaén, Spain; esiles@ujaen.es; 4 Berreich Organische Chemie, Martin Luther University Halle-Wittenberg, 06120 Halle (Saale), Germany; rene.csuk@chemie.uni-halle.de

**Keywords:** AKT/PI3K signaling pathway, apoptosis, human hepatocarcinoma HepG2 cells, migration activity, proliferation, oxidative stress, ROS level, uvaol

## Abstract

Natural products have a significant role in the development of new drugs, being relevant the pentacyclic triterpenes extracted from *Olea europaea L*. Anticancer effect of uvaol, a natural triterpene, has been scarcely studied. The aim of this study was to understand the anticancer mechanism of uvaol in the HepG2 cell line. Cytotoxicity results showed a selectivity effect of uvaol with higher influence in HepG2 than WRL68 cells used as control. Our results show that uvaol has a clear and selective anticancer activity in HepG2 cells supported by a significant anti-migratory capacity and a significant increase in the expression of HSP-60. Furthermore, the administration of this triterpene induces cell arrest in the G_0_/G_1_ phase, as well as an increase in the rate of cell apoptosis. These results are supported by a decrease in the expression of the anti-apoptotic protein Bcl2, an increase in the expression of the pro-apoptotic protein Bax, together with a down-regulation of the AKT/PI3K signaling pathway. A reduction in reactive oxygen species (ROS) levels in HepG2 cells was also observed. Altogether, results showed anti-proliferative and pro-apoptotic effect of uvaol on hepatocellular carcinoma, constituting an interesting challenge in the development of new treatments against this type of cancer.

## 1. Introduction

Cancer is one of the leading causes of mortality worldwide, especially in developed countries, on account of the aging of the population [1]. Liver cancer is the fifth most common type, as well as the second type of tumor that causes more deaths globally. Specifically, hepatocellular carcinoma (HCC) accounts for 90% of primary liver neoplasms, whose incidence increases progressively with ageing, according to the EASL (European Association for the Study of the Liver) [2].

Hepatocarcinogenesis is a process initiated by different external stimuli that induce genetic changes in hepatocytes or hepatic stem cells, which can cause alterations in the processes of proliferation and apoptosis, by dysfunctions in the cell cycle and its regulation, which eventually lead to tissue dysplasia and can cause a neoplasm [3]. In response to this DNA damage, different control points can be activated throughout the cell cycle phases. Among all the proteins that regulate this process, p53 stands out [4]. The activity blockage of protein kinases regulated by p53 produces an inhibition of cell cycle progression, due to an arrest in G_1_ phase [5]. It is known that p53 is frequently mutated in HCC, disrupting the correct role of this protein [6].

Another important pathway involved in hepatocarcinogenesis is the one controlled by c-*Myc* proto-oncogene. The elements of the *Myc* proto-oncogenes family have a role as strong transcription factors of other proteins that take part in the control of cell differentiation and proliferation, oncogenesis and apoptosis [7]. Studies show that c-*Myc* is overexpressed in HCC, in comparison with healthy patients [8]. 

One of the main characteristics of tumor cells is their resistance to cell death, so the apoptosis process is one of the most studied pathways. This programmed cell death can occur through two pathways: the mitochondrial or intrinsic and the one that involves death receptors, also named extrinsic. In response to various external stimuli, the mitochondria increases its permeability, releasing apoptotic mediators, among which proteins from the Bcl-2 family and cytochrome c stand out [9]. 

It is well established that NADPH molecules are essential in anabolic processes related to membranogenesis, as well as in nucleotide metabolism through ribose phosphate formation [10], and also plays a decisive role as a modulator of protein synthesis [11]. Therefore, the participation of NADPH in both metabolic aspects makes it especially important for growth and cell differentiation [12]. For these reasons, the regulation of NADPH levels is essential to understand the behavior of numerous physiological processes and, in this sense, nutritional conditions [13,14,15,16]; the presence of triterpenes [17,18]; and the redox state [19] modify significantly the levels of those reduction equivalents.

Reactive oxygen species (ROS) in non-pathological concentrations act as second messengers involved in several signal transduction pathways that regulate processes such as cell growth, proliferation and differentiation [20]. Therefore, cells have detoxification mechanisms that maintain a redox balance since, if they are altered, excessive production of ROS can lead to a situation of oxidative stress, which plays an important role in apoptosis and in the beginning of neoplasia development. Within these detoxification mechanisms, several enzymes stand out, such as superoxide dismutase (SOD), catalase (CAT) or glutathione peroxidase (GPX) [21].

Some pharmacological compounds used as HCC treatments, such as Sorafenib, inhibit VEGF angiogenic factor and MAPK pathway [22]. Since the efficacy of current therapies is low when advanced stages of HCC are considered, it is necessary to seek alternative treatments that could offer a better prognosis for patients [23]. Traditional medicine occupies an important place in the development of new drugs, since natural compounds are a recurrent source of molecules with bioactive properties [24].

The Mediterranean diet presents olive oil as its main exponent, which is obtained from the fruit of the *Olea europaea L.*. Its consumption is associated with various health benefits [24], among which there are a lower incidence of cardiovascular diseases, a lower production of ROS and even a reduction in the risk of cancer [25,26,27]. These properties are associated with their high content of monounsaturated fatty acids, with oleic acid being the most abundant, in addition to other minor compounds, such as tocopherols, polyphenols, triterpenes and squalene [28]. 

**Figure 1:**
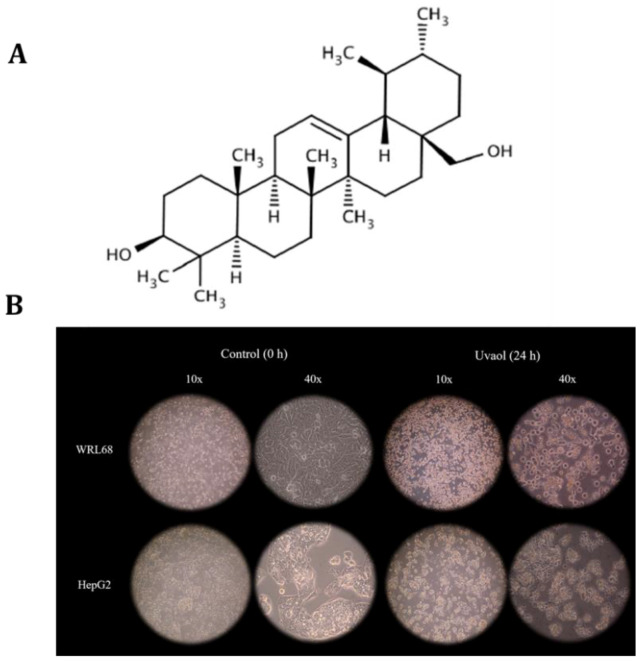
Chemical structure of uvaol (PubChem) (**A**). Morphological changes in response to uvaol treatment incubated at IC_50_ after 24 h in the WRL68 (IC_50_: 54.3 µg/mL) and HepG2 (IC_50_: 25.2 µg/mL) cell lines (**B**).

Uvaol (urs-12-ene-3,28-diol) is a natural alcohol pentacyclic triterpene whose formula and molecular weight is C_30_H_50_O_2_ and 442.73 g/mol, respectively. It has a structural isomer, erythrodiol, from which it only differs in the position of a methyl group, since it is located in carbon 19, while uvaol presents it in carbon 20. The most notable feature of this compound is that it exhibits two hydroxyl groups in remote positions, specifically in carbons 3 and 28 (Figure 1A) [29]. Among the described set of triterpenes present in olive oil, uvaol has the lowest characterization of its bioactive properties [30]. Martín et al. [31] studied the effect of uvaol on the 1321N1 cell line and found that uvaol increased the rate of apoptotic cells, as well as producing an activation of the JNK. Allouche et al. [32] studied the effects of uvaol on MCF-7 cells observing a decrease in ROS production and cell viability. 

Due to the promising results shown by the few studies carried out to date on the anticancer potential of uvaol, the aim of the present work was to evaluate its cytotoxic capacity, as well as its effect on proliferation, migration, morphology, cell cycle, apoptosis, oxidative stress, dual PI3K/MAPK signaling pathway and expression of protein markers involved in the processes described on the human control liver cell line WRL68 and the human hepatocellular carcinoma line HepG2.

## 2. Results

### 2.1. Uvaol Produces Morphological Changes in HepG2 Cells

Uvaol effect on cell morphology was analyzed using the IC_50_ concentration of each cell line for 24 h. The images taken at 10 and 40 magnifications of the four experimental conditions described are shown in Figure 1B. Referring to the WRL68 line, we can observe that its growth on the support is homogeneous, with the cells being initially separated from each other and presenting a fusiform morphology at 10 magnifications. When the treatment described is applied, the cells undergo a structure change, since they become rounded and have cytoplasmic projections towards the environment, due to cell death induced by the administration of uvaol. In addition, this causes them to rise from the support on which they grow.

The morphology of HepG2 cells in a control situation is triangular and shows an islet-shaped growth, each of them being well delimited with respect to the rest of the surrounding islets. Additionally, its cytoplasm is much more irregular than the presented by WRL68 cells. After the compound’s administration, as in the previous case, its characteristic morphology is blurred, becoming slightly more rounded, with very sharp projections and a loss of adhesion to the support. 

### 2.2. Uvaol Has a Selectivity Cytotoxic Effect on the HepG2 Cell Lines

**Figure 2:**
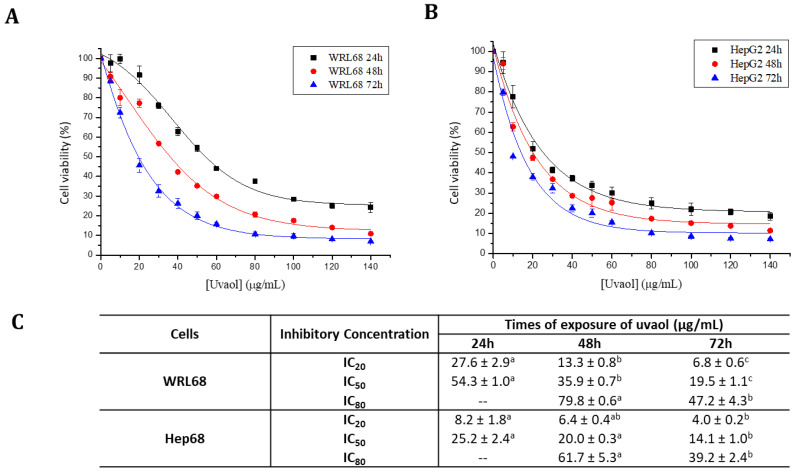
Cytotoxicity curves of uvaol during 24, 48 and 72 h in WRL68 (**A**) and HepG2 cells (**B**). IC_20_, IC_50_ and IC_80_ values for the WRL68 and HepG2 lines after 24, 48 and 72 h of incubation with uvaol (**C**). These values are represented by mean ± SEM (n = 9).

Uvaol effect was evaluated by the MTT assay on the WRL68 and HepG2 cell lines. The viability results obtained were represented in percentage (%) with respect to the different concentrations of compound administered (in μg/mL) for each cell line at 24, 48 and 72 h of exposure, showed using a sigmoidal adjustment (Figure 2A,B). Similarly, the IC_20_, IC_50_ and IC_80_ values were calculated in the three times lapses described (Figure 2C). Figure 2A shows that, for the WRL68 line, uvaol reduced cell viability in a dose and time-dependent way, since the IC_20_, IC_50_ and IC_80_ values decreased with this treatment in a progressive form for the three studied time lapses.

In the HepG2 line there is also a progressive decrease in cell viability according to concentration and exposure time (Figure 2B). The IC_50_ value obtained was 54.3 μg/mL (12.3 µM) for the WRL68 line and 25.2 μg/mL (5.7 µM) for the HepG2 line after 24 h of uvaol exposure (Figure 2C). Therefore, the concentration needed to inhibit cell proliferation in tumor cells was half that the needed for the same purpose on the control line. Following tests were carried out using the mentioned IC_50_ concentrations and a period of 24 h uvaol incubation. Differences between the IC_50_ values of both cell lines at later time points (48 h and 72 h) were diminished, but still statistically significant.

### 2.3. Anti-Migratory Activity of Uvaol in HepG2 Cells

**Figure 3:**
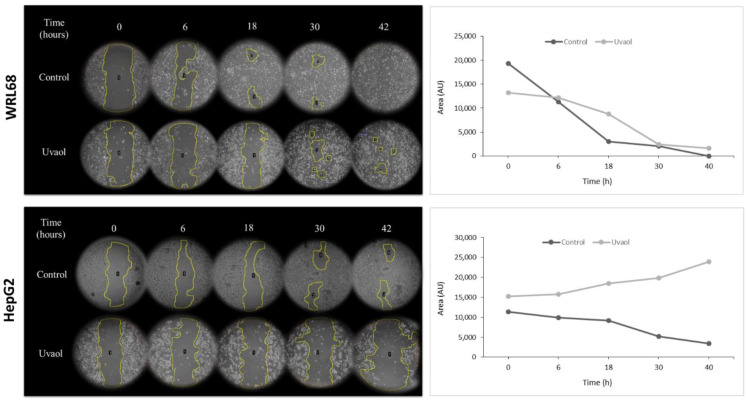
Representative image from wound healing assay of cells treated and no treated with uvaol at IC_50_ concentration (0, 6, 18, 30 and 42 h) in **WRL68** and **HepG2** cells. In the graph, quantification of the cell-free region is showed during the time of wound healing assay (AU: arbitrary units).

The wound healing assay (or scratch assay) aims to assess the ability of cancer cells to migrate in vitro. Figure 3 collects the several images taken and quantified in each experimental condition at 10 magnifications for 0, 6, 18, 30 and 42 h after the wound was made. Data in quantification for the WRL68 cell line (Figure 3) showed a progressive wound healing, occurring at 18 h, in the control situation, the junction of the separated areas. The complete closure occurred at 42 h. In the treated populations, the behavior of cells was different, since wound healing was maintained during more hours than the control group.

For the HepG2 line (Figure 3), in the control populations the wound was not totally closed at the end, although the values of the quantification showed a progressive closure of the wound. In the treated populations, the wound remains until the end of the trial.

### 2.4. Uvaol Induces G_0_/G_1_ Arrest in HepG2 Cells

**Figure 4:**
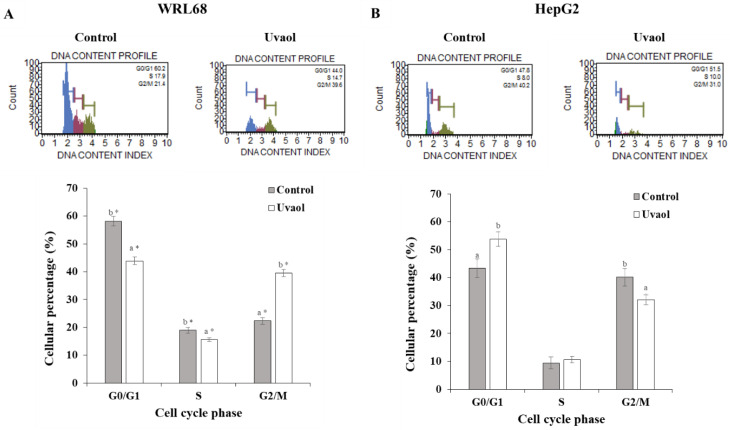
Cell cycle analysis obtained according to the Muse™ cell cycle kit. Panel (**A**) correspond to WRL68 cells and panel (**B**) to HepG2 cells. The cells were treated with IC_50_ of uvaol for 24 h. Top: histograms from a representative experiment show the effect of uvaol on cell cycle profile. Bottom: percentage of cells in each cellular cycle phase. Values are expressed as mean ± SEM (n = 12). Different letters indicate significant differences (*p* < 0.05) between control and uvaol treatment within each phases of the cell cycle for WRL168 or HepG2 cells. The inclusion of asterisks indicates significant differences (*p* < 0.05) between the different cells lines (WRL168 vs. HepG2), under the same treatment (control or uvaol) and phase of cell cycle.

In order to analyze the possible effect of uvaol in the cell cycle for each line studied, the proportion of cells that were in each phase of the cycle (G_0_/G_1_, S and G_2_/M) were measured in control and uvaol-incubation conditions. Figure 4 shows the images and quantifications of the cell cycle assay generated by the flow cytometer during its analysis for each experimental condition.

Uvaol produced a significant decrease in the percentage of cells in phase G_0_/G_1_ and phase S, while, on the contrary, induced an increase in the G_2_/M phase for the WRL68 line (Figure 4A). In the case of the HepG2 cell line (Figure 4B), treatment with uvaol resulted in a statistically significant increase in the percentage of cells that were in the G_0_/G_1_ phase, while causing an equally significant reduction of cells in G_2_/M phase. No differences were found in the S phase. When statistically comparing the results obtained between both lines, significant differences were observed in the behavior shown in each phase of the cell cycle after treatment with uvaol.

### 2.5. Apoptosis Is Enhanced in HepG2 Cells by Uvaol

**Figure 5:**
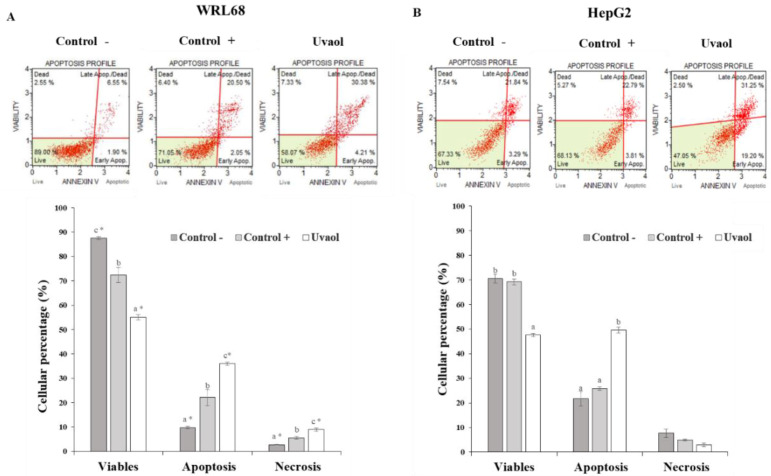
Apoptosis analysis obtained according to the Muse™ apoptosis kit. Panel (**A**) corresponds to the WRL68 cells and panel (**B**) to the HepG2 cells. Treatments included cells not treated (negative control) and cells incubated with staurosporine (1 μg/mL, positive control) or IC_50_ of uvaol for 24 h. Top: dot plots show a representative experiment of the different treatments. Bottom: percentage of lived, apoptotic and necrotic cells for each treatment. Values are expressed as mean ± SEM (n = 12). Different letters indicate significant differences (*p* < 0.05) between control and uvaol treatment within apoptosis stage (viable, apoptosis or necrosis) for WRL168 or HepG2 cells. The inclusion of asterisks indicates significant differences (*p* < 0.05) between different cells lines (WRL168 vs. HepG2), under the same treatment (control or uvaol) and apoptosis stage.

Apoptosis assay provides information about the type of cell death that occurs in each line and for each situation studied: negative control, positive control (treatment with staurosporine with a 1 μg/mL concentration for 2 h) and treatment with uvaol. Figure 5 includes the images of each experimental condition generated by the flow cytometer during its analysis. The data obtained reflect the percentage of viable cells, those that suffer apoptosis and those presenting necrosis (Figure 5).

Figure 5A shows how both the addition of staurosporine (control +) and uvaol produced a significant decrease in the number of viable cells in the WRL68 line, which was accompanied by an equally significant increase in the number of apoptotic and necrotic cells in each situation with respect to control. In HepG2 cells (Figure 5B), treatment with staurosporine did not lead to an increase in apoptotic cells, while uvaol did induce a significant reduction in the percentage of viable cells, therefore, increasing the proportion of apoptotic cells. In none of the situations studied, changes in the rate of necrotic cells were generated.

When statistically comparing the results in both cell lines, significant differences were found in the death rate generated by uvaol in each case. The percentage of cells in apoptosis is higher in the HepG2 line, while, on the contrary, a higher rate of necrosis occurred in the WRL68 line, although significantly lower than the number of cells in apoptosis. Therefore, uvaol exerts a pro-apoptotic effect on both lines.

### 2.6. Uvaol Decreases ROS Production in HepG2 Cells

**Figure 6:**
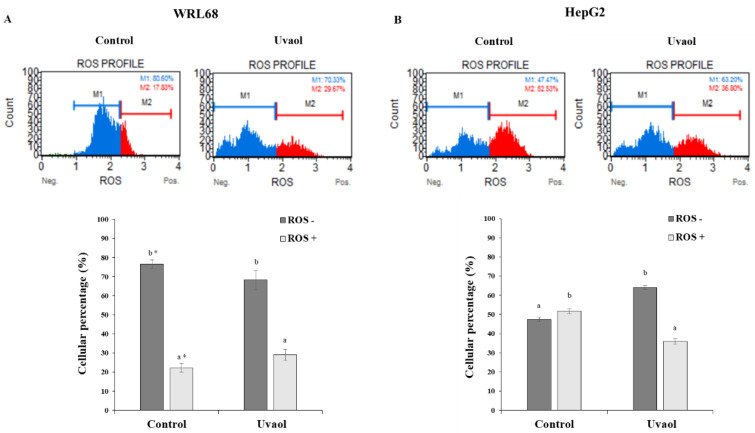
Reactive oxygen species (ROS) quantification was performed according to the Muse™ Oxidative stress kit. Panel (**A**) corresponds to the WRL68 cells and panel (**B**) to the HepG2 cells. Treatments included cells not treated and cells incubated with IC_50_ of uvaol for 24 h. Top: dot plots show a representative experiment of the different treatments. Bottom: percentage of ROS negative (ROS−) and ROS positive (ROS+) values observed for each treatment. Values are expressed as mean ± SEM (n = 12). Different letters indicate significant differences between ROS levels for each treatment and an asterisk indicates significant differences between cell lines for each treatment and ROS levels (*p* < 0.05).

Intracellular ROS levels were measured by FACS under the four generic test conditions described above. Figure 6 includes the images of each experimental condition generated by the flow cytometer during its analysis. The results show the percentage of cells in each situation studied that express ROS (ROS+) and those that do not express them (ROS−).

Uvaol treatment maintained the ROS levels produced by the WRL68 cell line without significant differences with respect to the control situation (Figure 6A). The highest levels of ROS were produced in the control situation in the HepG2 cell line (Figure 6B). When treated with uvaol, a significant decrease in the percentage of cells expressing this type of reactive species was observed. When statistically comparing the results in both cell lines, no significant differences in ROS levels between the WRL68 and HepG2 lines after treatment were found. In control situation, significant differences were observed in ROS production levels, since HepG2 express higher levels of radicals. 

### 2.7. Uvaol Produces Up and Down-Regulation of Target Proteins in HepG2 Cells

**Figure 7:**
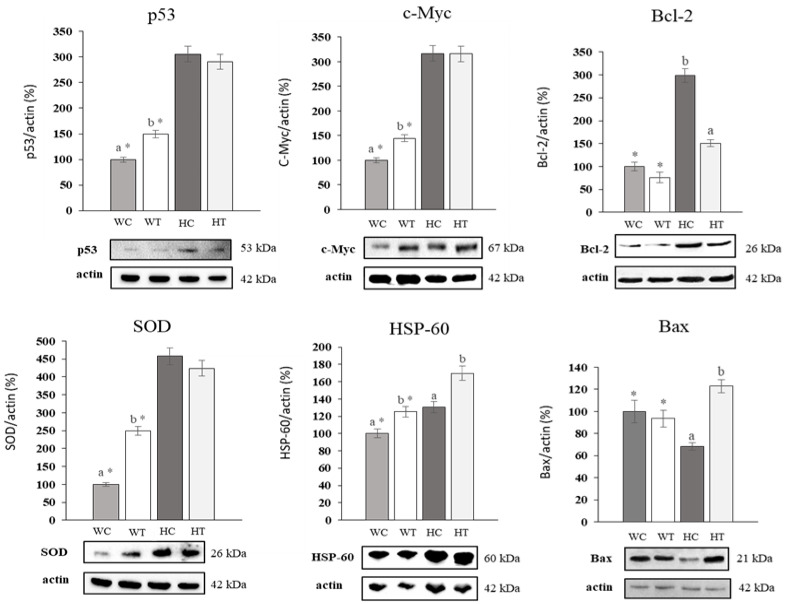
Western-blot analysis of **p53**, **c-Myc**, **Bcl-2**, **SOD**, **HSP-60** and **Bax** protein levels in WRL68 and HepG2 cells, untreated (WC and HC) and exposed to IC_50_ of uvaol for 24 h (WT and HT). The quantification of protein levels by densitometric analysis is shown in bar graphs. The results are the means ± SEM (n = 9) and are expressed as percentage of expression compared to actin. WRL68 values were used as 100% of expression and the rest of treatments were referred to them. Different letters indicate significant differences between treatments for each cell line and an asterisk indicates significant differences between cell lines for each treatment (*p* < 0.05).

The expression of p53, c-Myc, Bcl-2, SOD, HSP-60 and Bax proteins for the WRL68 and HepG2 cell lines, in control situation and after treatment with the corresponding IC_50_ concentration of uvaol for 24 h, was measured through the Western blot technique. The level of expression obtained in each case was normalized with respect to the expression of actin (constitutive protein) and subsequently referred to that obtained in the control situation for the WRL68 line (Figure 7).

The expression of p53 increased in the WRL68 line after treatment with uvaol regarding control, but remained unchanged in HepG2 cells, a situation that is repeated for SOD. In both cases, the percentage of control expression and after treatment for the HepG2 line was higher, with respect to expression after treatment in WRL68. In the case of c-Myc, an increase in its expression was observed in WRL68 with respect to the control, while it was maintained in HepG2. C-Myc levels in the control situation in HepG2 cells were higher than its expression after treatment in WRL68. The expression of HSP-60 increased in both lines when applying the treatment, being the percentage of expression after the addition of uvaol in WRL68 equal to the control situation in HepG2. Finally, the treatment did not produce changes in the expression of Bcl-2 either Bax in WRL68 cells, while their expression levels decreased and increased in HepG2 cells, respectively. In all the cases, the protein expressions were higher in the HepG2 cells than in the WRL68 cells when compared under the same experimental conditions.

### 2.8. Uvaol Modulate Dual PI3K/MAPK Signaling Pathway in HepG2 Cells

**Figure 8:**
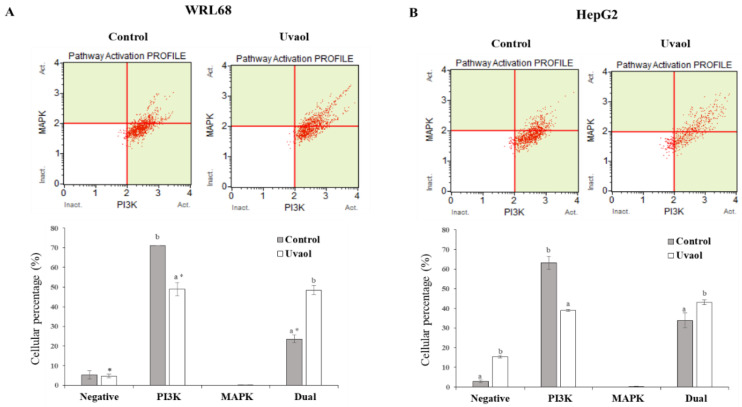
AKT/PI3K and ERK/1/2/MAPK dual pathway determination was performed according to the Muse™ PI3K/MAPK Dual Pathway activation kit. Panel (**A**) correspond to WRL68 cells and panel (**B**) to HepG2 cells. Treatments included cells not treated and cells incubated with IC_50_ of uvaol for 24 h. Top: dot plots show a representative experiment of the different treatments. Bottom: percentage of non-activated cells and cells with PI3K, MAPK and Dual pathway activated for each treatment. Values are expressed as mean ± SEM (n = 12). Different letters indicate significant differences between treatments for each activated pathway and an asterisk indicates significant differences between cell lines for each treatment and activated pathway (*p* < 0.05).

To analyze the mechanism by which uvaol exerts its anticancer activity, we studied whether such effect could be produced through the dual PI3K/MAPK signaling pathway. The results are shown in Figure 8. Uvaol treatment produced a 22% decrease in cells that only activated the AKT/PI3K pathway, whereas the percentage of cells in which only ERK1/2/MAPK was activated, besides being too low, was not altered in the WRL68 cells. However, an increase of 25% was observed in cells that activated the dual pathway (MAPK and PI3K) (Figure 8A). When a net balance of activated signaling pathways is considered, no modification of the PI3K pathway was observed, but an activation of 25% took place in the ERK1/2/MAPK signaling pathway. 

In HepG2 cells, uvaol effect on the percentage of cells that only activated AKT/PI3K induced a decrease of 25%. Similar to WRL68 cells, the percentage of cells that only activated ERK1/2/MAPK, besides being too low, was not altered in HepG2 cells. Regarding the activated dual pathway (MAPK and PI3K), uvaol only produced a 9% increase (Figure 8B). The net balance, in HepG2 cells, resulted in an inhibition of 16% in AKT/PI3K signaling pathway, whereas ERK1/2/MAPK signaling pathway was only increased in 9%.

## 3. Discussion

The Mediterranean diet is mainly characterized by the regular intake of olive oil, which presents in its composition different molecules with beneficial properties for health. Among them, pentacyclic triterpenes, such as uvaol, have been shown to have an anti-parasitic, anti-oxidant, anti-inflammatory and hepatoprotective activity, especially highlighting their potential as anticancer molecules [18]. Due to the lack of effective treatments against cellular hepatocarcinoma in advanced stages [33], and the fact that no researches about effect of uvaol have been performed in these type of cancer, this study was intended to characterize the anticancer activity of uvaol in the human cell lines WRL68 (hepatic control) and HepG2 (cellular hepatocarcinoma). For this purpose, its effect on the proliferation, migration, morphology, cell cycle, apoptosis, oxidative stress levels and protein markers of the processes described on the liver lines mentioned were determined.

The cytotoxicity assay is a useful test to preliminarily detect compounds that can affect the number of healthy cells in a total population. This trial showed that uvaol exerts an inhibition of cell proliferation in a dose and time dependent manner. Especially noteworthy is the result obtained for the 24 h IC_50_ concentration in both cell lines, being 25.2 μg/mL and 54.3 μg/mL for the HepG2 and WRL68 lines, respectively. As it can be seen, the concentration required to inhibit 50% of proliferation in control cells was more than twice the one used to inhibit the proliferation of tumor cells at the same rate, indicating greater susceptibility and specificity to the compound by hepatocarcinoma cells. To our knowledge, studies of the uvaol in cancer cells are limited. In this sense, similar results were observed in the human mammary tumor line MCF-7 in which uvaol produced anti-proliferative effects in a dose and time-dependent manner with values IC_50_11.06 μg/mL and 44.27 μg/mL [32]. Other triterpenes olive derived, such as maslinic acid, have been tested in HepG2 cells, in which the cytotoxic effect was lower to that observed in this study, with a IC_50_ of 47 μg/mL at 72 h [34]. These values demonstrate a greater cytotoxic effect of uvaol than maslinic acid. Moreover, oleanolic-type saponins lowered the growth and proliferation of HepG2 in transplanted tumor in mice [35].

Triterpenes present in olive oil, such as ursolic acid or oleanolic acid have demonstrated anti-migratory and anti-angiogenic activities on several tumor lines [18]. To study if uvaol also had this effect, a wound healing assay was performed in the HepG2 cell line, confirming that this compound has an anti-migratory effect. Moreover, this effect was corroborated by a simultaneous morphological characterization, since it was observed that, after the addition of the compound, cells presented alterations in their structure and adhesion ability. Uvaol induced these same changes on the human astrocytoma line 1321N1, making cells show a loss of adhesion [31]. Furthermore, the administration of oleanolic acid in HepG2 caused a cellular retraction and the appearance of cytoplasmic extensions characteristic of the apoptosis process [36].

Some natural triterpenes exert anti-proliferative effects, due to their interference with the progression of the cell cycle or by the induction of cell death through apoptosis [30]. The arrest of the cycle in some of its phases constitutes a defense mechanism that allows damage repair in DNA, characteristic of tumoral cells [4]. Likewise, apoptosis is an essential process in organisms, since it allows for the elimination of cells, in a controlled manner, whose behavior or characteristics are away from homeostasis [5]. For this reason, flow cytometry studies were conducted to determine the process involved in the cytotoxic effect of uvaol on the cell lines under study. The results obtained showed that this triterpene was capable of inducing cell death through apoptosis with the corresponding concentration of IC_50_ in both cell lines. Similar results were found in lymphoma cells (U937), in which 10 and 100 µM of uvaol induced apoptosis [32]. Notwithstanding, the same concentrations of uvaol did not show this effect in MDA-MB-231 (triple negative breast cancer) or in MCF-7 cells [32]. Uvaol-induced cell cycle arrest occurred in the G_0_/G_1_ phase for HepG2 cells, while in WRL68 it was in the G_2_/M phase. The use of uvaol on the MDA-MB-231 line did not produce changes in the progression of the cycle [29], while a concentration of 44.27 μg/mL of uvaol on the MCF-7 line and of 22.11 μg/mL of oleanolic acid on the HepG2 line resulted in an arrest in the same phase of the cycle [32,36], results that match with those obtained. Therefore, the anti-proliferative effect of uvaol on HepG2 cells is due to, simultaneously, the induction of apoptosis and an arrest in the G_0_/G_1_ phase of the cell cycle.

In cancer initiation, ROS levels are usually increased, due to the generation of a pro-oxidant environment maintained over time. This situation of oxidative stress, generally induced by an exacerbated production of free radicals, or by an imbalance in endogenous cellular antioxidant systems, can lead to the production of DNA damage, an initial step in neoplastic development [21]. For this reason, controlling ROS levels inside a cancer cell could be a good strategy to mitigate the genetic material damage, or even prevent to it. Triterpenes are characterized by having an antioxidant activity [18], a quality manifested in the HepG2 results obtained in the present study. Similar results were obtained in the MCF-7 and MDA-MB-231 lines for uvaol and erythrodiol [29,32]. However, these compounds produced an increase in the intracellular levels of ROS in 1321N1 cells, which also resulted in the reduction of mitochondrial potential and in an induction of apoptosis via the JNK kinase pathway [31]. Therefore, the action of uvaol on the oxidative state of cells is specific to each tumor cell line. Nevertheless, during normal detoxification process of any foreign compound, such as uvaol inside the cell, ROS can be also produced [37]. An augmentation of ROS levels in response to uvaol treatment did not occur in WRL68 cells, probably due to the increase of SOD expression found in the present study.

p53 and c-Myc proteins are directly involved in the regulation of cell proliferation, as well as in the succession of the different phases of the cell cycle and in the apoptosis process [5,7]. For this reason, the expression profile of the mentioned proteins was characterized in the WRL68 and HepG2 cell lines. Contrary to expectations, p53 levels were higher in the HepG2 than in WRL68 cells. This may be due to the fact that, despite being described a decrease in p53 expression in patients with HCC, due to mutations at different points of the gene that encodes it (TP53) [8], the HepG2 line has this gene intact, being probably induced its expression by situations far from homeostasis in cancer cells, such as an increase in ROS intracellular levels, appreciated in our results. Due to the cellular arrest observed in the G_0_/G_1_ phase and the maintenance of p53 expression levels after treatment with uvaol in HepG2 cells, it is possible that the inhibition of cell cycle progression is on account of the alteration of the expression of other proteins involved in this process, such as the family of Cdks or their inhibitors, among which are p15, p16, p21 or p27 [5]. Similarly, the increase in c-Myc expression caused by treatment in the WRL68 line may be related to the higher rate of cells found in the G_2_/M phase, since c-Myc presents an important proliferative function. One of the main activities of c-Myc is cell cycle control, since it has been descripted that c-Myc expression levels tightly correlate to cell proliferation. Indeed, c-Myc is in charge of inducing the expression of several positive regulators of the cell cycle, such as Cdk4/6, E2F, Cyclin E, Cyclin A, Cdk1/2, among other factors. C-Myc also represses several cell cycle inhibitors, such as p15, p16, p21 or p27 [38]. Overall, it can be stated that an overexpression of c-Myc correlates with an increase percentage of proliferating cells, that is, cells in G2/M phase, as observed in treated WRL68 cells. In the case of the HepG2 line, the increase found in the control situation for c-Myc falls within the expected range, since it is a cancer cell with a high proliferation capacity. This same expression profile was found by Koutb et al. [7] when analyzing the gene expression of the c-Myc gene in blood samples obtained from HCC patients.

The family of proteins that share BH domains contribute to the progression of the cell cycle and the induction of apoptosis, since they are involved in survival mechanisms and in pathways that prevent cell proliferation. The most important member of this family is Bcl-2, a protein with anti-apoptotic effects that is generally increased in certain types of neoplasms, among which is HCC, giving them greater invasive ability and a lower response to treatments [39]. In our study, we observed how Bcl-2 levels remained unchanged after treatment with uvaol in the WRL68 line. As expected, its expression was increased in untreated HepG2 cells with respect to the control line. The administration of uvaol produced a decrease in the levels of this anti-apoptotic protein in the cancer line, a result that is consistent with the induction of cell death due to apoptosis, and the arrest in the G_0_/G_1_ phase obtained in our findings. Other triterpenes have been reported to also induce apoptosis in HepG2 cells through a down-regulation in Bcl-2 [40]. Similar results have been also found in other cancer cell lines, such as HT29 [41]. Moreover, these authors also observed that Bax pro-apoptotic protein expression increased in response to treatment with maslinic acid [41]. These results are in concordance to the results observed in the present study in which uvaol treatment increased Bax in HepG2 cells. 

Heat shock proteins (HSP) form a family with a fundamental role in the correct folding and functionalization of proteins synthesized inside the cell. Clinical studies in patients with HCC show that the expression of HSP-60 is diminished in tumor cells with respect to healthy livers. This finding is related to a greater invasive ability of this neoplasm and a lower survival rate in patients. The lower differentiation gives these cells greater mobility, resulting in a high rate of invasion and subsequent metastasis [42]. Our results show that uvaol treatment induces an increase in the expression of HSP-60 in the WRL68 control line, being this rise more acute for the HepG2 line. This effect of uvaol in the HCC lines is a promising result, since it is related to the results obtained by Zhang et al. [42]. These authors observed that after inducing an overexpression of the gene that codes for the HSP-60 in HepG2 cells, these cells developed a phenotype with lower migratory capacity and greater cell differentiation, thus, decreasing their metastatic ability [42]. Together with HSP-60 overexpression, wound healing results obtained in this study confirm the effect of uvaol as a potential anti-migratory compound. 

The AKT/PI3K pathway is known to be one of the most important signaling routes, which participates in cell growth, proliferation, cellular apoptosis and cytoskeletal rearrangement [43,44]. AKT is the major downstream target of the AKT/PI3K pathway. In addition to its interaction with Bcl-2 family effectors, the survival signal to cells is transferred by phospho-AKT [45]. Many studies have demonstrated that this pathway is activated in several types of cancer [46]. The results of the present study showed that uvaol significantly decreased AKT/PI3K pathway in HepG2 cells, whereas no changes were observed in the WRL68 line. On the contrary, levels of ERK1/2/MAPK were increased in both lines, although this increment was significantly lower in hepatoma cells. This up-regulation on MAPK pathway was consequence of the down-regulation on PI3K, since PI3K and Ras pathways can intersect by cross-talk among their downstream effectors [47]. In other HCC studies (HepG2, Huh-7, Hep3B, and Sk-Hep-1 cell lines) using triterpenoids, such as ursolic acid or platycodin D, the results also showed an inhibition of AKT/PI3K signaling pathway [48,49,50]. These results support the hypothesis that apoptosis induced by uvaol is mediated by the modulation of the AKT/PI3K signaling pathway in HepG2 cells. 

Considering the promising in vitro preliminary results of uvaol usage as an anticancer compound (anti-proliferative, pro-apoptotic and antioxidant) drawn from the present work, our future research will be focused on testing these same bioactive properties of uvaol in additional HCC cell lines, with the aim to finally move into in vivo preclinical models. Our next objective is to create HCC cell line-derived xenografts (CDX) and patient derived xenografts (PDX), to see if the observed antitumor properties of uvaol are maintained in vivo. These observations could provide enough evidence for considering a future usage of uvaol in humans as a nutraceutical compound in the food industry or even a therapeutic drug in the clinic; current milestones in cancer research. 

## 4. Materials and Methods 

### 4.1. Cell Cultures 

The cell lines used were WRL68 (model liver cells) and HepG2 (hepatocellular carcinoma), provided by “*Centro de Instrumentación Científica*” (CIC) of the University of Granada. The cells were grown in Dulbecco′s modified Eagle medium (DMEM) and minimum essential medium (MEM), respectively. In both cases, they were supplemented with 10% heat-inactivated foetal bovine serum (FBS) and 1% streptomycin/penicillin antibiotics. They were kept in a CO_2_ incubator at 37 °C, 95% relative humidity and 5% CO_2_. Cells were passaged at preconfluent densities by the use of a solution containing 0.05% trypsin and 0.5 mM EDTA. The cells were seeded in the culture dishes at the desired density with the appropriate culture medium.

### 4.2. Uvaol Solution

The compound tested in the experiments performed was uvaol, provided by Sigma^®^ (St. Louis, MO, USA), with a purity greater than 95%. A stock solution of uvaol with a concentration of 1 mg/mL (in 40 µL of DMSO + 960 µL culture medium) was prepared and subsequently diluted in culture medium until reaching the concentrations required for each test.

### 4.3. Morphological Changes

A morphological characterization of both cell lines in triplicate was carried out in a control situation (cells growing attached in culture medium) and after uvaol treatment corresponding to the IC_50_ calculated for 24 h. Optical microscopy (Olympus^®^ model CX22LED, Tokyo, Japan) images of the cultures were obtained in the mentioned conditions at 10 and 40 magnifications for the cell line each after 24 h of the treatment’s application.

### 4.4. MTT Assay

MTT assay was performed as described by Pérez-Jiménez et al. [51]. Briefly, samples containing 200 µL cell suspension (1 × 10^4^ cells/well) were cultured in 96 well plates, in triplicate of three populations of the both cell lines. Subsequent to adherence of the cells within 12 h of incubation, Uvaol was added to the wells at a concentration between 0–140 μg/mL and maintained during 24, 48 and 72 h. MTT was dissolved in the medium and added to the wells at a final concentration of 0.5 mg/mL. Following 2 h of incubation, the generated formazan was dissolved in DMSO. Absorbance was measured at 570 nm in a multiplate reader (Bio-tek^®^, Winooski, VT, USA). The concentrations that caused 20%, 50% and 80% of inhibition of cell viability (IC_20_, IC_50_ and IC_80_) were calculated following the formula: % cell viability = (A0 − AT)/A0·100, where A0 is the control absorbance (100% of cell viability) and AT is de absorbance of the incubated cells with the different concentrations of uvaol. OriginPro 8 (OriginLab Corporation, Northampton, MA, USA) was used to performance a dose-response analysis by the following formula:

$$y=A_{1}+\;\frac{A_{2}-\;A_{1}}{1+10^{\left(Log\;x_{0}-x\right)p}}$$

In which $Logx_{0}$ is the center of the curve, *p* the slope, $A_{1}$ the lower asymptote and $A_{2}$ the upper asymptote in the adjustment model described.

### 4.5. Scratch Assay

The wound healing or scratch assay was used to assess the migration capacity of the WRL68 and HepG2 cell lines. With this objective, 3 × 10^5^ cells were seeded per well in a 6-well plate in triplicate for each cell line. When the populations reached a confluence of 80–90%, a sterile pipette tip was used to disrupt in a straight line (make a wound) the layer of cells attached to the culture surface. Subsequently, it was washed with PBS to prevent the cells that had been lifted from being able to rejoin. The control and monitoring of cell migration was carried out by imaging with optical microscopy the culture at different times (0 h, 6 h, 18 h, 30 h and 42 h) at 10 magnifications from the realization of the wound, to be able to identify the possible differences between the control cases (growing cells attached in culture medium) and after the addition of the IC_50_ concentration for 24 h in both lines. Quantification of wound healing was performed by using the MRI-Wound Healing Tool for ImageJ software (ImageJ 1.53a version).

### 4.6. Cell Sorting by Flow Cytometry

A total of 1.5 × 10^5^ cells were seeded per well in 24-well plates for each cell line (quadruplicates for three different populations of each cell line). After 24 hallowing the cells to adhere, they were separated into four different groups: negative control WRL68 and HepG2 populations and WRL68 and HepG2 populations treated with the concentration corresponding to their uvaol IC_50_ for 24 h (in addition to other specific conditions of each test described in the corresponding section). Negative control populations were incubated only with the appropriate culture medium. The samples were analyzed in the Muse^TM^ Cell Analyzer (Merck-Millipore^®^, Burlington, MA, USA).

#### 4.6.1. Cell Cycle Assay

The method is based on the analysis of the cell cycle through the discrimination of three populations: cells in phase G_0_/G_1_, S or G_2_/M. For this, the Muse^TM^ Cell Cycle Kit purchased from Millipore (Billerica, MA, USA) was used following the specification of manufacturer.

#### 4.6.2. Apoptosis Assay 

Phosphatidylserine (PS) is a phospholipid that is usually found in the inner half of the cytoplasmic membrane, but is outsourced when loss of integrity occurs in the apoptosis process. The method is based on the recognition and binding to the PS exposed by annexin V protein, labeled with a fluorescent substance to detect the interaction and quantify it. We used the Muse^TM^ Annexin V & Dead Cell kit purchased from Millipore (Billerica, MA, USA), which provides the percentage of viable cells, apoptotic and affected by necrosis. A positive control was carried out, incubating cells with staurosporine (1 μg/mL), a nonspecific inhibitor of kinase proteins isolated from the *Streptomyces staurosporeus* species, which shows the ability to induce the apoptotic pathway in a large number of tumor lines, being especially characterized this effect on the HepG2 cell line [35].

#### 4.6.3. Measurement of ROS Production

The method is based on the use of dihydroetide (DHE), a reagent capable of crossing the cell membrane and interacting with superoxide anions, a type of ROS. In this process, DHE oxidizes and forms the DNA intercalating agent ethidium bromide, detectable by its fluorescence emission. This allows two cell populations to be distinguished, those that express remarkable levels of ROS (ROS+) and those that do not (ROS−). The Muse^TM^ Oxidative Stress kit purchased from Millipore (Billerica, MA, USA) was used for this purpose.

### 4.7. Protein Extraction and Western Blot Analysis

Triplicates of three different populations of each cell line, WRL68 and HepG2, were seeded in 6-well plates with a density of 2.5 × 10^5^ cells per well. The corresponding IC_50_ concentration for 24 h was added in the treated populations. For protein extraction, each sample was resuspended in 20 μL of RIPA buffer (150 mM NaCl, 1% Igepal, 0.5% deoxycholic acid, 0.1% SDS, 50 mM Tris HCl pH 7.5, 0.2 M PMSF, 700 mM OV_4_ and Thermo Scientific^®^ PierceTM inhibitor cocktail, Waltham, MA, USA), and held on ice for 15 min. They were centrifuged at 15,000× *g* for 10 min at 4 °C, the supernatant was collected and stored at −80 °C for further analysis. The quantification of the protein concentration was carried out through the Bradford method. 

The methodology used for Western blot analysis is described by Mokhtari et al. [19]. Brie fly, the initial separation of the extracts was performed by a 12% polyacrylamide gel electrophoresis under denaturing conditions (SDS-PAGE). A standardized load of 30 μg of protein per well was used after denaturing the samples at 95 °C for 5 min and adding the loading buffer (0.25 mM Tris-HCl pH 6.8, 10% SDS, 1 M DTT and glycerol). The proteins were transferred, by the semi-dry transfer method, (60 mA per gel, 1 h) using the TransBlot Turbo system (BioRad^®^, Berkeley, CA, USA), to a PVDF membrane. The membranes were blocked with blocking buffer (TBS, 0.1% Tween-20 and 3% skimmed milk) for 1 h at room temperature, incubating with the specific primary antibodies against the proteins studied at 4 °C overnight. The primary antibodies (from Santa Cruz Biotechnology^®^, Dallas, TX, USA) were diluted in blocking buffer. The membranes were washed with TBS-T (TBS, 0.1% Tween-20) for 5 min three times under stirring, and incubated with the corresponding secondary antibody (from Sigma^®^, St. Louis, MO, USA) (Table 1) for 1 h at room temperature, repeating the previous washing process later. These secondary antibodies have the enzyme horseradish peroxidase (HRP) coupled, which allows chemiluminescence to be detected in the presence of the protein band due to its ability to oxidize the luminol solution (ECL-plus Western-blot detection system, GE Healthcare, Chicago, IL, USA). The reaction produced was captured inside a ChemiDoc Imaging System (Bio-Rad^®^, Berkely, CA, USA). The program used to analyze the images obtained was Image Lab Software (Bio-Rad^®^, for PC 6.1 version). The expression of each protein was normalized referring to actin levels and were represented based on the results obtained for the control situation (WRL68 line without treatment) as percentage of expression (%). The expression obtained in each protein was analyzed through the mentioned program in triplicate.

### 4.8. PI3K/MAPK Dual Pathway Activation Assay

The Muse^®^ PI3K/MAPK Dual Pathway activation kit purchased from Millipore (Billerica, MA, USA) was employed to examine both the PI3K and MAPK signaling pathways simultaneously using the Muse Cell Analyzer (Merck-Millipore^®^, Burlington, MA, USA). The protocol followed was performed as the manufacturer instructions. The samples (quadruplicates for three different populations of each cell line) were analyzed in the Muse^TM^ Cell Analyzer.

### 4.9. Statistical Analysis

The results were expressed as mean ± standard error of the mean (SEM). Likewise, the study of the statistical difference between the data groups was carried out through the analysis of the two-way variance (two-way ANOVA). When interactions between the analyzed factors were present, the one-way analysis of the variance (one-way ANOVA) was used followed by the Tukey test. The differences were considered significant for *p* values < 0.05. Statistical analyses were performed using the IBM SPSS Statistics (IBM^®^, 22.0 version) software.

## 5. Conclusions

In conclusion, taken together, the results of this study suggest that uvaol exhibits a potential anti-proliferative effect through G_0_/G_1_ cell cycle arrest, apoptosis, by inhibition of AKT/PI3K signaling pathway, and decreased ROS levels on the HepG2 human hepatocarcinoma cell line.

## Figures and Tables

**Table 1:** Description of the polyclonal antibodies (IgG) used in the Western blot technique.

Protein	Primary Antibody	Secondary Antibody
**p53**	Rabbit anti-p53 antibody (1:500)	Anti-mouse IgG antibody (1:5000)
**SOD**	Rabbit anti-SOD antibody (1:250)	Anti-rabbit IgG antibody (1:5000)
**c-Myc**	Goat anti-c-Myc antibody (1:500)	Anti-goat IgG antibody (1:5000)
**HSP-60**	Mouse anti-HSP-60 antibody (1:250)	Anti-mouse IgG antibody (1:5000)
**Bcl-2**	Rabbit anti-Bcl-2 antibody (1:500)	Anti-rabbit IgG antibody (1:5000)
**Bax**	Mouse anti-Bax antibody (1:500)	Anti-mouse IgG antibody (1:5000)
**Actin**	Mouse anti-actin antibody (1:1.000)	Anti-mouse IgG antibody (1:5000)
